# Morphological, Thermal, and Mechanical Assessment of Polypropylene and Ammonium Phosphate Composites Enhanced with Lignosulfonate and Zirconium

**DOI:** 10.3390/polym16192727

**Published:** 2024-09-26

**Authors:** Keiti Gilioli Tosin, Cesar Aguzzoli, Matheus Poletto

**Affiliations:** 1 Postgraduate Program in Process Engineering and Technologies, University of Caxias do Sul, Caxias do Sul 95000-000, Brazil; kgtosin@ucs.br; 2 Graduate Program in Materials Science and Engineering, University of Caxias do Sul, Caxias do Sul 95000-000, Brazil; caguzzol@ucs.br

**Keywords:** composites, polypropylene, ammonium phosphate, lignosulfonate, zirconium phosphate, magnetron sputtering

## Abstract

Polypropylene and ammonium phosphate (AP) composites were synthesized at a 25 wt% concentration. The changes in the morphological, thermal, and physical behavior of the composites were analyzed with the addition of lignosulfonate (LG) and zirconium phosphate (ZrP). Additionally, metallic zirconium was deposited onto lignosulfonate using the magnetron sputtering technique to develop polypropylene and zirconium-modified lignosulfonate (LGMod) composites. Thus, composites of PP/25AP, PP/25AP/8LG/5ZrP, and PP/25AP/8LGMod were synthesized. The synthesis involved mixing the materials in a Hake mixer, followed by compression molding. The composites were characterized by field emission scanning electron microscopy (SEM–EDS), a thermogravimetric analysis (TGA) with combustion parameters, a vertical burn test (UL-94), a thermal camera, and mechanical properties. All composites achieved a V2 rating according to UL-94 standards. The PP/25AP extinguishes flames more quickly compared to other materials, approximately 99.2% faster than PP and showed the lowest temperature variation and mass loss after burning. The PP/25AP/8LG/5ZrP composite exhibited a 7% higher rigidity and 84.5% better flame retardancy compared to pure PP. Additionally, substituting ZrP with LGMod led to a lower environmental impact and improved thermal properties, despite some mechanical disadvantages.

## 1. Introduction

Mechanical and thermal resistance, rigidity, electrical insulation, and the stability of a composite are influenced by the type, quantity, and arrangement of the reinforcement present in the matrix. The reinforcement can modify the characteristics of the material, and the greater the amount of reinforcement, the higher the product’s resistance. In this way, it is possible to manufacture composites with properties superior to some polymers [1]. Composites with thermal properties, such as flame retardancy, have been studied with the aim of replacing halogenated materials [2]. Non-halogenated flame retardants are environmentally friendly, cost-effective, have char-forming capabilities, and have a low toxicity [3]. However, the advancement of these materials still faces several challenges, such as their limited efficacy, which often requires substantial volumes of the compound to achieve satisfactory results, impacting the material properties [4]. The combination of nitrogen and phosphorus elements, for example, has the potential to create effective flame-retardant synergy but compromises the mechanical properties of the polymer [5]. Therefore, it is essential to carefully consider the choice of these additives [6].

Lignosulfonate is a byproduct of lignin, a compound that has been used as a flame retardant due to its thermal stability and reactivity to radicals, both in the gaseous and condensed phases [7]. Furthermore, the chemical modification of lignin has been recognized as an effective strategy to enhance its thermal stability and performance as a flame-retardant agent [8]. Lignosulfonate also shows the potential to act as a flame-retardant agent due to its aromatic structure and the presence of sulfonate, hydroxyl, phenolic, and carboxyl groups [9]. These groups possess properties that facilitate the formation of chemical bonds and interactions with material surfaces, creating a protective layer [10]. Moreover, lignosulfonate can function as an acid and serve as a carbon supplier in the system, accelerating the development of more stable char residues and reducing the combustion rate [11]. It is essential to note that incorporating lignosulfonate into polymers can also enhance the mechanical properties of the blend [9].

In recent years, there has been an increased interest in polymeric composites incorporating an inorganic phase due to their ability to provide mechanical strength, thermomechanical resistance, gas barrier properties, and flame retardancy while preserving the lightweight and optical transparency of the polymer [12]. The incorporation of zirconium ions into polymers has the potential to create a protective layer that delays flame propagation and enhances the thermal stability of the composite [13]. One of the main properties of zirconium is its resistance to corrosion, especially in highly acidic and alkaline environments. The metal also has high mechanical strength and rigidity, as well as low thermal and electrical conductivity, making it useful in applications that require heat or electricity transfer [14]. Crystalline zirconium phosphate has been applied in flame-retardant polymeric composites due to its thermal resistance and chemical stability characteristics [15]. When zirconium phosphate is used as a flame-retardant agent in polymers, it undergoes a loss of H+ ions, resulting in the exposure of acidic protons in its degraded layers. These acidic protons catalyze the cross-linking of polymeric chains, leading to the formation of char residues during the combustion process [16]. In this way, zirconium phosphate increases the resistance of the char layer and acts as a catalyst in the carbonization of polypropylene at high temperatures [17].

Another alternative for utilizing zirconium can be through deposition. According to Li et al. [18], metal deposition can be carried out through various methods, including physical routes. The advantage of these routes is that they minimize environmental impacts, as there is no need for chemicals in the process. The magnetron sputtering technique is a material deposition process that involves the sputtering of atoms from a solid target to deposit them onto a substrate surface [19]. Although metal nanoparticles do not naturally possess flame-retardant properties, their addition in small quantities to polymeric composites generally leads to a noticeable improvement in thermal stability, reduced smoke emissions, control of the maximum heat release rate, and deceleration of flame propagation in nanocomposites [20].

The use of materials with a lower environmental impact is a current challenge that requires research to assess the feasibility of replacing synthetic materials with more sustainable alternatives [21]. Furthermore, most studies use chemical routes for synthesizing polymer composites, leading to the generation of pollutants. Therefore, there is a recognized need for the development of the present study.

## 2. Materials and Methods

The materials used included polypropylene (PP), sodium lignosulfonate (LG), zirconium (Zr), and zirconium phosphate (ZrP). The selected PP is a copolymer provided by Braskem (São Paulo, Brazil), grade CP 442 XP, with a melt flow index of 6 g/10 min (230 °C/2.16 kg). The LG, commercially known as Ultrazine NA, was supplied by Borregaard Linotech (São Paulo, Brazil), and ZnP and ZnO_2_ were provided by Sigma Aldrich (Barueri, Brazil). In addition, the LG modified with zirconium was deposited with a Zr target from Williams Advanced Materials (Buffalo, New York, NY, USA) with a purity of 99.5%. The materials used were dried in an oven at 80 °C for approximately 4 h.

### 2.1. Modification of Lignosulfonate

The deposition of zirconium nanoparticles was carried out using the cathodic sputtering method (or sputtering), utilizing the magnetron sputtering equipment located in the Surface Engineering and Heat Treatments Laboratory (LESTT) at the University of Caxias do Sul (UCS). The employed method involves the ejection of atoms from a zirconium target surface through the bombardment of energetic argon ions, resulting in the deposition of zirconium onto the lignosulfonate substrate. The base pressure of the chamber was 10^−5^ mbar. The deposition parameters were set to 120 W of power at the target, 30 min of deposition time, and a pressure of 4 × 10^−3^ mbar during deposition.

### 2.2. Sample Preparation

For the composite mixing, a Haake torque rheometer (Thermo Scientific, Caxias do Sul, Brazil) was used. Initially, the materials were processed at a rotation speed of 60 rpm at 180 °C for 5 min. The composites were molded into plate shapes using a compression molding process at a temperature of 180 °C and a pressure of 10 tons for 5 min. Afterward, the composites were subjected to a cold press with a pressure of 6 tons for approximately 5 min. The test specimens were obtained by cutting the plates, which were subsequently used in mechanical tests. In this way, seven samples were prepared, as represented in Table 1. A 25 wt% ammonium phosphate content was selected for the composites [22]. The selection of lignosulfonate and zirconium phosphate contents was determined to be 8 wt% and 5 wt%, respectively, based on the studies by Lu et al. (2023) and Chen et al. (2021) [17,23].

### 2.3. Composite Characterization

The composition of the lignosulfonate was analyzed using energy dispersive spectroscopy (EDS). This technique involves the use of an X-ray detector to identify the elemental composition of a material. In this case, a silicon drift detector (SDD) from Oxford Instruments (High Wycombe, United Kingdom), specifically the X-act model, was employed.

Qualitative analyses of the composites were conducted using field emission scanning electron microscopy (FEG–SEM) with a Tescan microscope—model FEG Mira 3 (Tescan mira, Brno, Czech Republic) along with energy dispersive spectroscopy (EDS) to assess the dispersion of additives in the polymer matrix. A thermal gravimetric analysis (TGA) was essential to evaluate the thermal stability of the developed composites. The experiment was conducted in a nitrogen (N_2_) atmosphere with a gas flow of 50 mL/min, covering a temperature range from 23 °C to 600 °C and a heating rate of 10 °C/min. This analysis was performed using Shimadzu (Japan) TGA-50 equipment (Kyoto, Japan) with a platinum crucible.

The results obtained through thermogravimetry were used to determine the mass loss rate and the different phases of degradation, allowing for the analysis of the combustion parameters of the samples. The parameters considered include ignition temperature (T_i_), complete burning temperature (T_B_), combustion index (S), ignition index (D_i_), the time corresponding to the maximum degradation rate (t_m_), ignition time (t_ig_), maximum degradation rate, and average degradation rate. According to Protásio et al. (2019) [24], the ignition temperature is defined as the point at which the thermal degradation rate increases by 1% per minute, marking the beginning of the primary thermal degradation process. Conversely, the complete burning temperature is the point at which the thermal degradation rate decreases by 1% per minute, indicating the end of the thermal degradation process. Additionally, the time corresponding to the maximum degradation rate and the ignition time are determined from the respective maximum degradation and ignition temperatures.

The combustion index (S) was calculated using Equation (1), as referenced by Moon et al. (2013) [25] and Protásio et al. (2019) [24]. The ignition index (D_i_) was determined using Equation (2), following the method proposed by Xiang-Guo et al. (2006) [26]. These parameters correspond to the mass variation over time at the point of maximum degradation (max) and the average mass variation over time (average) throughout the entire thermal degradation process.

(1) $$P=\frac{\left(\frac{dm}{dt}\right)\left(\frac{dm}{dt}\right)me}{T_{i}^{2}\;X\;T_{B}}$$

(2) $$D_{i}=\frac{\left(\frac{dm}{dt}\right)max}{T_{m}\;X\;T_{ig}}$$

The vertical burning test was conducted following UL-94 standard [27] guidelines to assess the flame spread of the composites. The analysis was conducted in quintuplicate. A Teledyne FLIR LLC—T300 series (Teledyne FLIR, Wilsonville, OR, USA) thermal camera was used during the UL-94 test. The images were captured after the first and second burns of the samples. Three focal points were selected and adjusted according to the extent of the specimens. The analysis was performed in triplicate. Furthermore, mechanical tests were performed to assess the impact of additives on PP. The flexural strength tests were conducted according to ASTM D790-17 [28] with five specimens. The test speed was 1.5 mm/min. Additionally, impact strength tests were performed using the unnotched Izod method with 10 specimens, according to ASTM D256-10 [29]. The tensile tests were conducted according to ASTM D638-03 with a speed of 5 mm/min.

## 3. Results and Discussion

The compositions of lignosulfonate (LG) and lignosulfonate modified with zirconium (LGMod) determined by energy dispersive spectroscopy (EDS) are presented in Table 2. According to theoretical calculations, the hydrogen (H) content is estimated to be approximately 4% by weight. The type of wood used for extracting lignosulfonate can significantly influence its final structure and composition, leading to variations in the chemical composition, with substantial differences particularly between hardwood and softwood species [30].

Despite the low concentration of zirconium, its presence can significantly impact the properties of lignosulfonate, depending on how it interacts with the material’s structure and the specific requirements of the application. Even in small amounts, the addition of zirconium can be advantageous, especially if the application requires improvements such as enhanced thermal stability or barrier properties [20]. Musl et al. (2021) [30] investigated the composition of a commercial hardwood lignosulfonate obtained from the same supplier as that analyzed in the present study. They found 2.18 mmol/g of aliphatic groups, 2.28 mmol/g of aromatic groups, and 0.18 mmol/g of carboxylic groups. In comparison, the lignosulfonates analyzed in this study show similar levels of aliphatic and aromatic groups, but with a slight increase in carboxylic groups in the modified lignosulfonate (5.0 ± 0.4% for LGMod versus 4.5 ± 0.5% for LG), which may indicate a difference in the sulfonation process or the addition of zirconium. This modification could suggest potential alterations in the lignosulfonate’s chemical properties and functionality, potentially enhancing its performance in specific applications.

**Figure 1:**
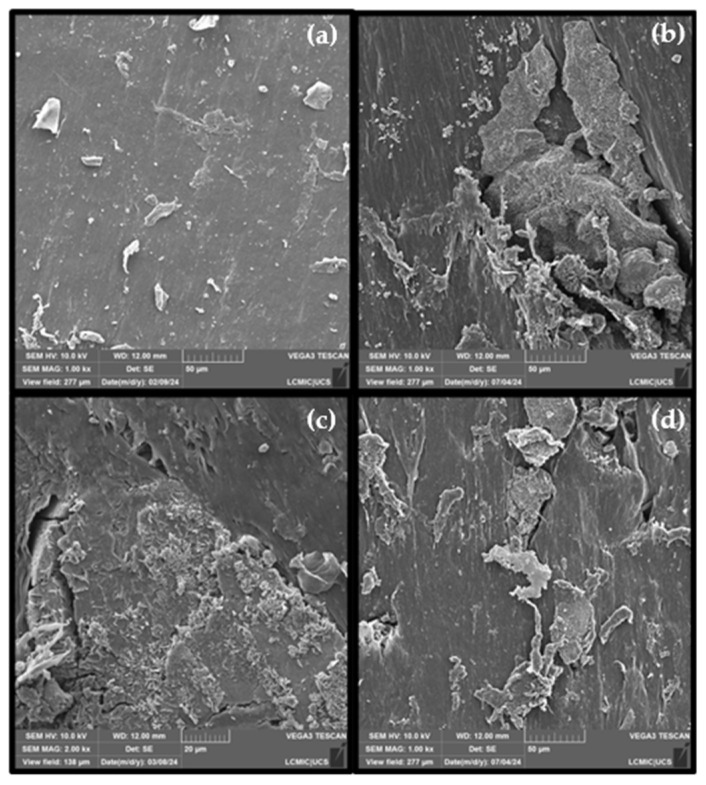
Micrographs at a resolution of 1000× of (**a**) PP, (**b**) PP/25AP, (**c**) PP/25AP/8LG/5ZrP, and (**d**) PP/25AP/8LGMod.

The morphologies of the synthesized composites at a magnification of 1000× are presented in Figure 1. In Figure 1a, the morphology of PP is shown, where points with roughness and characteristic irregularities on the surface of the material can be observed. When analyzing images Figure 1b–d, where the compounds were incorporated, a homogeneous distribution of the particles within the polymer matrix is observed, indicating that the components were incorporated into the matrix. Additionally, it was observed that the composites maintained their structural integrity, with no signs of agglomeration or phase separation. This occurs because the presence of phosphate groups can improve the adhesion of other materials, as it promotes the formation of a compatible interface between the reinforcing material and the polymer matrix, which is crucial for the mechanical strength and durability of the composites.

**Figure 2:**
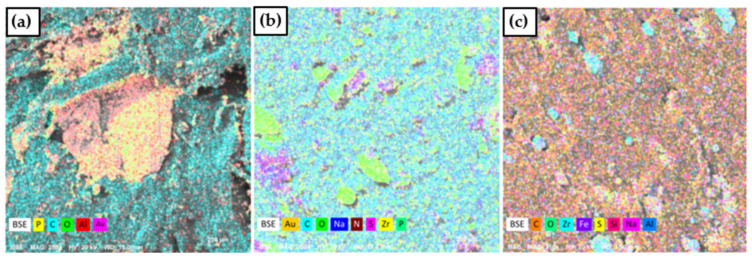
EDS of (**a**) PP/25AP, (**b**) PP/25AP/8LG/5ZrP, and (**c**) PP/25AP/8LGMod.

Figure 2 shows the energy dispersive spectroscopy images of the composites. It is evident that when only ammonium phosphate was used in combination with PP, phosphate agglomeration occurred (a). On the other hand, when lignosulfonate and zirconium phosphate were added, the phosphate was able to disperse more effectively, although some agglomeration remained (b). This can be explained by the incorporation of lignosulfonate, which also acts as a dispersing agent [31]. Figure 2c provides clear evidence that zirconium was successfully incorporated into the lignosulfonate, as represented in the EDS analysis.

**Figure 3:**
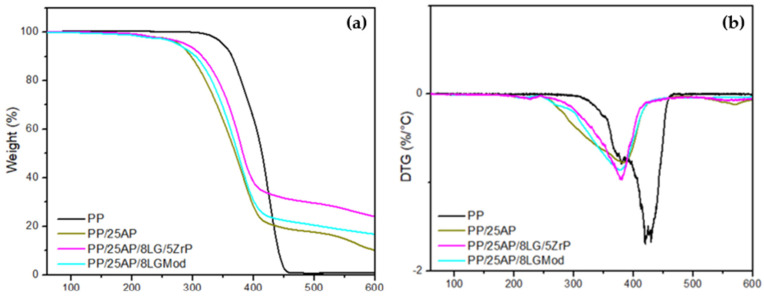
(**a**) TG and (**b**) DTG of PP and the developed composites.

The thermogravimetric analysis of PP and the composites is presented in Figure 3a along with its derivative Figure 3b. PP exhibits two stages of oxidation, with the first stage occurring from 300 °C to ~375 °C and the second from 375 °C to 500 °C. It can be observed that the degradation profile shifts to higher temperatures with the increase in heating rate, consistent with the results from other researchers [32]. The temperature at which PP showed the highest rate of mass loss (430 °C) is comparable to the temperatures reported by Ciliz et al. (2004) [33] at 458 °C for virgin PP and by Valle et al. (2004) [34] at 457 °C. The composites exhibited three stages of mass loss, with the first stage between 186 °C and 246 °C, the second from 253 °C to 420 °C, and the third around 525 °C to 593 °C. The first peak may be related to the decomposition of organic additives or the release of volatile components present in the composites. The third peak, on the other hand, can be attributed to the decomposition of more stable structures or the formation of new decomposition products at high temperatures. According to Cavdar et al. (2019) [35], the maximum mass loss temperature for composites with AP can be attributed to the release of water and ammonia. Moreover, the incorporation of phosphate with lignin induces thermal destabilization below 400 °C and improves stability once this temperature is exceeded. Additionally, the greater reduction in the mass loss rate in the presence of oxygen can be attributed to phosphorus, which is known to prevent the oxidation of char [8].

Table 3 presents the results for the temperature at which a 3% mass loss occurs, the maximum degradation temperature (T_peak_), and the ash content at 600 °C. The composites with ammonium phosphate began to lose mass around 260 °C and had a char formation temperature of approximately 377 °C. Although the thermal stability of the composites was shown to be lower compared to PP in Figure 3, the residuals were higher. In the article by Prieur et al. (2016) [8], the material with the highest residual ash content also achieved the best flame retardancy results in a calorimetric mass loss test.

The combustion parameters were calculated for the materials in Table 4. Protassio et al. (2019) [24] indicate that lignin, the source of lignosulfonate, plays a crucial role in the combustion process. While elevated levels of volatile matter can cause mass loss in the early stage of combustion, the subsequent stage is characterized mainly by the combustion of solid carbon. In this phase, lower amounts of volatile matter were associated with increased mass loss. The combustion index (S) and the ignition index (Di) are fundamental parameters for assessing a material’s ability to resist combustion, as well as its susceptibility to ignition. A decrease in these indices indicates greater effectiveness of the material as a flame retardant, reducing the risk of ignition and fire spread [25]. The PP/25AP/8LGMod composite showed a reduction in these parameters compared to pure PP of approximately 25% and 14%, respectively. These results were very similar to those of the PP/25AP/8LG/5ZrP composite, indicating that the modification of lignosulfonate is a viable alternative. Additionally, the PP/25AP composite also exhibited lower combustion and ignition indices compared to the pure polymer, suggesting that all the developed materials may have potential applications as flame retardants.

Table 5 presents the results obtained for the composites in the vertical burning test according to UL-94. The pure polymer was able to extinguish the flames during the first burn but went into full combustion during the second burn. For a polymeric material to receive a V2 classification in the vertical burn test, it must exhibit an individual burn time of less than 30 s and a total burn time of less than 250 seconds for the five test specimens used [36].

All the developed composites were able to self-extinguish the flames and achieved a V2 flame retardant classification according to UL-94. This result corresponds with the combustion parameters calculated in Table 4, where these same materials had the lowest ignition and combustion indices. Among the composites, the PP/25AP sample was the one that managed to extinguish the flames most quickly, with a burn time of 0.9 seconds for five test specimens and 0.4 seconds for each individual specimen. The PP/25AP/8LGMod composite had a burn time of 18.3 seconds for five test specimens and 9.1 seconds for each specimen. Compared to PP/25AP/8LG/5ZrP when LGMod is added instead of ZrP, it is possible to self-extinguish the flames approximately 78% faster.

**Figure 4:**
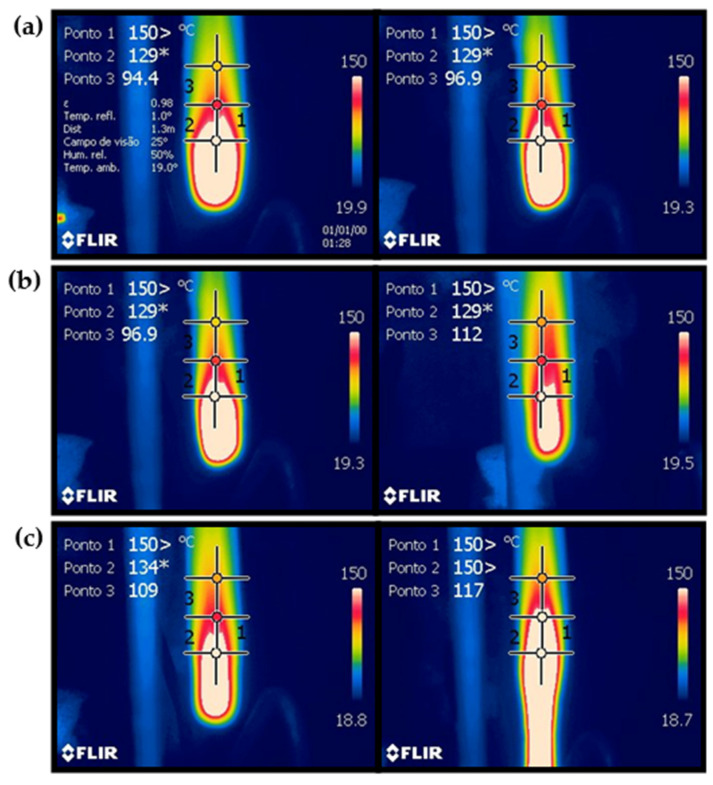
Thermal images obtained after the first and second burns, respectively, for the composites (**a**) PP/25AP, (**b**) PP/25AP/9LG/5ZrP, and (**c**) PP/25AP/8LGMod.

The thermal images captured by the camera are presented in Figure 4 for (a) PP/25AP, (b) PP/25AP/8LG/5ZrP, and (c) PP/25AP/8LGMod, with the images taken during the first and second burns, respectively. The temperature data analysis at various points provides insight into the thermal uniformity of the composites under heating conditions. In all composites, the temperatures at point 1 exceeded the thermal camera’s limit, making accurate measurement impossible. Point 3 showed the greatest variation among the composites. For example, the PP/25/8LGMod sample reached higher temperatures even at a distance from the ignition source, while the PP/25AP sample had lower temperatures at the selected points, leading to a lower overall average.

For the PP/25AP composite (a), it is observed that there was little temperature variation between the first and second burns, as this material tends to self-extinguish the combustion process quickly, as seen in the UL-94 test shown in Table 5. Due to this, heat concentrated at the ends of the samples caused the third point captured by the thermal camera to show lower temperatures compared to the other composites. This material experienced only a 3.27% mass loss after burning, demonstrating its high thermal stability and minimal degradation under combustion conditions. The PP/25AP/8LG/5ZrP material (b) showed similar temperatures at the three fixed points. The highest temperatures were recorded in the center of the sample, likely due to increased heat concentration and reduced thermal dissipation in that area, while the edges exhibited the lowest temperatures. The mass loss after burning was 16.28% of the material. In contrast, the PP/25AP/8LGMod composite exhibited a more significant difference between the first and second burns. The second burn revealed a larger area with higher temperatures and noticeable edge deformation. However, the material only lost 7.50% of its mass, suggesting the composite’s structural integrity was largely preserved despite the deformation.

**Figure 5:**
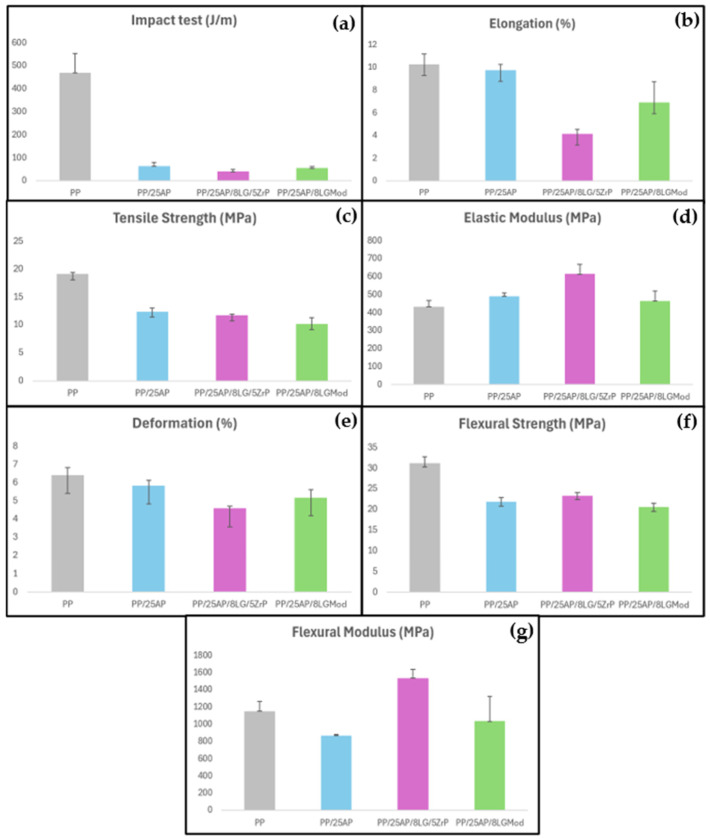
(**a**) Impact testing, (**b**) elongation, (**c**) tensile strength, (**d**) elastic modulus, (**e**) deformation, (**f**) flexural strength, and (**g**) flexural modulus of the materials.

The mechanical properties of the materials are shown in the graphs presented in Figure 5. Overall, a decrease in mechanical properties is observed when flame retardant fillers are added to PP. This may occur due to a lack of compatibility between the additives and the polymer matrix, despite the good distribution of the elements, as shown in Figure 1.

In Figure 5a, it is seen that pure PP was the material that showed the highest values in the impact test, while the developed composites exhibited similar values. The addition of ammonium phosphate can cause a decrease in impact resistance due to the heterogeneity introduced into the polymer matrix. These additives can act as stress concentration points or alter the toughness of the material, resulting in lower energy absorption during impact compared to pure PP. Regarding elongation (b), it is seen that the PP/25AP composite showed similarity to PP, while the samples with lignosulfonate exhibited lower values. According to Wang et al. (2015) [37], lignin is a polar material with limited compatibility with non-polar synthetic polymers, such as PP, leading to a decline in the mechanical properties of various polymer blends. All composites also showed lower tensile strength (c) and flexural strength (f) parameters compared to the virgin polymer. 

The limited compatibility between the additives and the PP matrix can create weak interfaces, which compromise the structural integrity of the composite under tensile and flexural loads. In contrast, the developed composites also showed a higher elastic modulus (d) compared to PP, indicating that the reinforcements were able to create a more robust internal network that resists deformation under loads. This behavior can be explained because a more robust internal reinforcement network can improve mechanical properties such as strength and stiffness by better filling the voids in the matrix and making the material more rigid. However, if the reinforcements do not align correctly with the PP matrix, they may cause weaknesses and compromise the mechanical properties of the material. The PP/25AP/8LG/5ZrP composite was the material that exhibited the highest elastic modulus and the highest flexural modulus (g) among the others. Thus, it is evident that zirconium phosphate contributes to increasing the material’s rigidity. This fact explains why the same composite shows greater deformation compared to the other materials analyzed in (e). In this case, the other composites showed values close to those of pure PP.

## 4. Conclusions

It was confirmed by SEM that the additives were incorporated into the PP matrix. A thermogravimetric analysis (TGA) assessed the combustion and ignition indices, revealing that the composites with the lowest values also performed better in the vertical burn test according to UL-94. Thus, the materials PP/25AP, PP/25AP/8LG/5ZrP, and PP/25AP/8LGMod achieved a V2 rating and can be considered flame retardants. The PP/25AP composite extinguishes flames more quickly (0.9 seconds for five specimens) compared to PP/25AP/8LGMod (18.3 seconds for five specimens). The substitution of ZrP with LGMod allows for flame self-extinguishment approximately 78% faster. Thermal images revealed that the PP/25AP composite exhibited the lowest temperature variation between fires and a mass loss of 3.27% after burning, indicating high thermal stability and rapid flame extinction.

In terms of elongation, PP/25AP performed similarly to pure PP, while the composites with lignosulfonate showed lower elongation. For impact resistance, pure PP outperforms the composites, but the composites exhibit a higher elastic modulus, indicating greater rigidity. Among the composites, PP/25AP/8LG/5ZrP displays the highest elastic modulus and flexural modulus, indicating that this composite has the greatest rigidity and resistance to deformation. Although pure PP offers better impact performance, the developed composites, particularly PP/25AP/8LG/5ZrP, show significant improvements in rigidity and flame retardancy properties, expanding their potential applications in various contexts. The use of LGMod compared to ZrP has proven to be beneficial, as it demonstrated better thermal properties despite some inferior mechanical parameters. Additionally, this substitution contributes to a lower environmental impact.

## Figures and Tables

**Table 1:** Encoding and quantification of PP and synthesized composites.

Ecoding	PP (%) *	AP (%) *	LG (%) *	ZrP (%) *	LGMod (%) *
PP	100	0	0	0	0
PP/25AP	75	25	0	0	0
PP/25AP/8LG/5ZrP	62	25	8	5	0
PP/25AP/8LGMod	67	25	0	0	8

* % m/m, AP = ammonium phosphate, LG = lignosulfonate, LGMod = modified lignosulfonate, and ZrP = zirconium phosphate.

**Table 2:** Composition of pure lignosulfonate and zirconium-modified lignosulfonate.

Samples	C (w%)	O (w%)	Na (w%)	S (w%)	Zr (w%)
LG	57.4 ± 1.8	32.7 ± 1.7	5.2 ± 0.3	4.5 ± 0.5	0.0
LGMod	58.3 ± 0.2	30.6 ± 0.5	5.2 ± 0.1	5.0 ± 0.4	0.9 ± 0.1

**Table 3:** Data obtained from thermal analyses for PP and the composites.

Samples	T_3% ML_ (°C)	T_peak_ (°C)	Ash Content (600 °C) (%)
PP	341	430	0.8
PP/25AP	261	377	9.8
PP/25AP/8LG/5ZrP	263	380	24.0
PP/25AP/8LGMod	254	376	16.7

**Table 4:** Combustion parameters of PP and the composites.

Samples	T_i_ (°C)	T_B_ (°C)	t_ig_ (min)	t_m_ (min)	$\left(\frac{dm}{dt}\right)$_max_ (%/min)	$(\frac{dm}{dt}{)}_{\mathrm{average}}$ (%/min)	S × 10^−7^ (%^2^min^−2^ °C^−3^)	D_i_ × 10^−2^ (%min^−3^)
PP	330	457	31.5	41.2	15.4	1.7	5.28	1.19
PP/25AP	249	441	22.5	35.5	7.5	1.5	4.13	0.94
PP/25AP/8LG/5ZrP	281	421	25.5	32.5	9.5	1.4	3.98	1.15
PP/25AP/8LGMod	261	438	23.6	35.0	8.5	1.4	3.97	1.02

**Table 5:** Vertical burn test of PP and the composites.

Samples	t1 ^a^/t2 ^b^ (s)	Total Burn	Dripping	Classification
PP	14.2/110.6	Sim	Sim	-
PP/25AP	3.0/3.0	Não	Sim	V2
PP/25AP/8LG/5ZrP	19.0/7.0	Não	Sim	V2
PP/25AP/8LGMod	18.0/1.2	Não	Sim	V2

^a^: maximum combustion time of 5 samples after the first 10 s ignition; ^b^: maximum combustion time of 5 samples after the second 10 s ignition.

## Data Availability

Data are contained within the article.
